# Retroperitoneal Fibrosis following Etanercept for Ankylosing Spondylitis

**DOI:** 10.31138/mjr.181025.erf

**Published:** 2026-06-09

**Authors:** Su Li Goh, Deepak Nagra, Madusha M. Jayasinghe, Rofaida Hassan, Maryam Adas, Mariam Baghaffar, Asad Khan, Eleni Stathopoulou, Mona Musa, Mary Gayed, Sujata Ganguly, Sophia Khan, Arvind Sinha

**Affiliations:** 1 Department of Rheumatology, Solihull, Birmingham, United Kingdom;; 2 Centre for Rheumatic Disease, King’s College London, London, United Kingdom;; 3 Centre for Rheumatic Disease, University Hospitals Birmingham NHS Foundation Trust, Birmingham, United Kingdom

**Keywords:** ankylosing spondylitis, etanercept, spondyloarthropathy

To the Editor,

With the expanding use of biologic and targeted synthetic disease-modifying antirheumatic drugs (DMARDs), paradoxical immune-mediated reactions are being recognised with increasing frequency across rheumatology and other medical specialties. They can mimic true vasculitides and arthritides questioning the fine line between autoimmunity and autoinflammatory pathology. Paradoxical reactions are used to describe the effect of a drug approved for a specific indication when the use of this otherwise therapeutically active substance induces the opposite of what is intended, the recurrence of non-infectious inflammation, or the exacerbation of a predisposed disease.^1^ This reaction does not fit in neatly within either innate or adaptive immune cascades, and no current biomarkers are identified to aid diagnostic processes.

To date, the use of monoclonal antibody therapies has been increasingly reported with this phenomenon, whereas other targeted agents as Janus Kinase Inhibitors (JAKis) being less frequently implicated.^2^ Mechanistically, monoclonal antibodies- for example, adalimumab- have been linked to type 1 interferon imbalance that may underlie this paradoxical effect, This effect is less frequently reported with JAK inhibitors, which can concurrently inhibit interferon signalling pathways.^3^ Secondly, the blockade of tumour necrosis factor (TNF) receptor-2 is associated with a loss of self-tolerance and T regulatory cell dysfunction,^4^ which is thought to be linked to the overall process of paradoxicality.^4^ Case reports have documented the occurrence of such reactions with the use of monoclonal antibody treatments.^5^

We present the case of a 44-year-old of South Asian descent who was initially diagnosed with radiographic ankylosing spondylitis ten years prior who presented with bilateral loin pain. She was treated with Adalimumab (40mg subcutaneous injection every two weeks) for 2 years before switching to Secukinumab, an interleukin 17A inhibitor, (150mg monthly) due to worsening disease. She remained on Secukinumab for 4 months before switching to Etanercept due to primary inefficacy in disease control.

**Figure 1.**
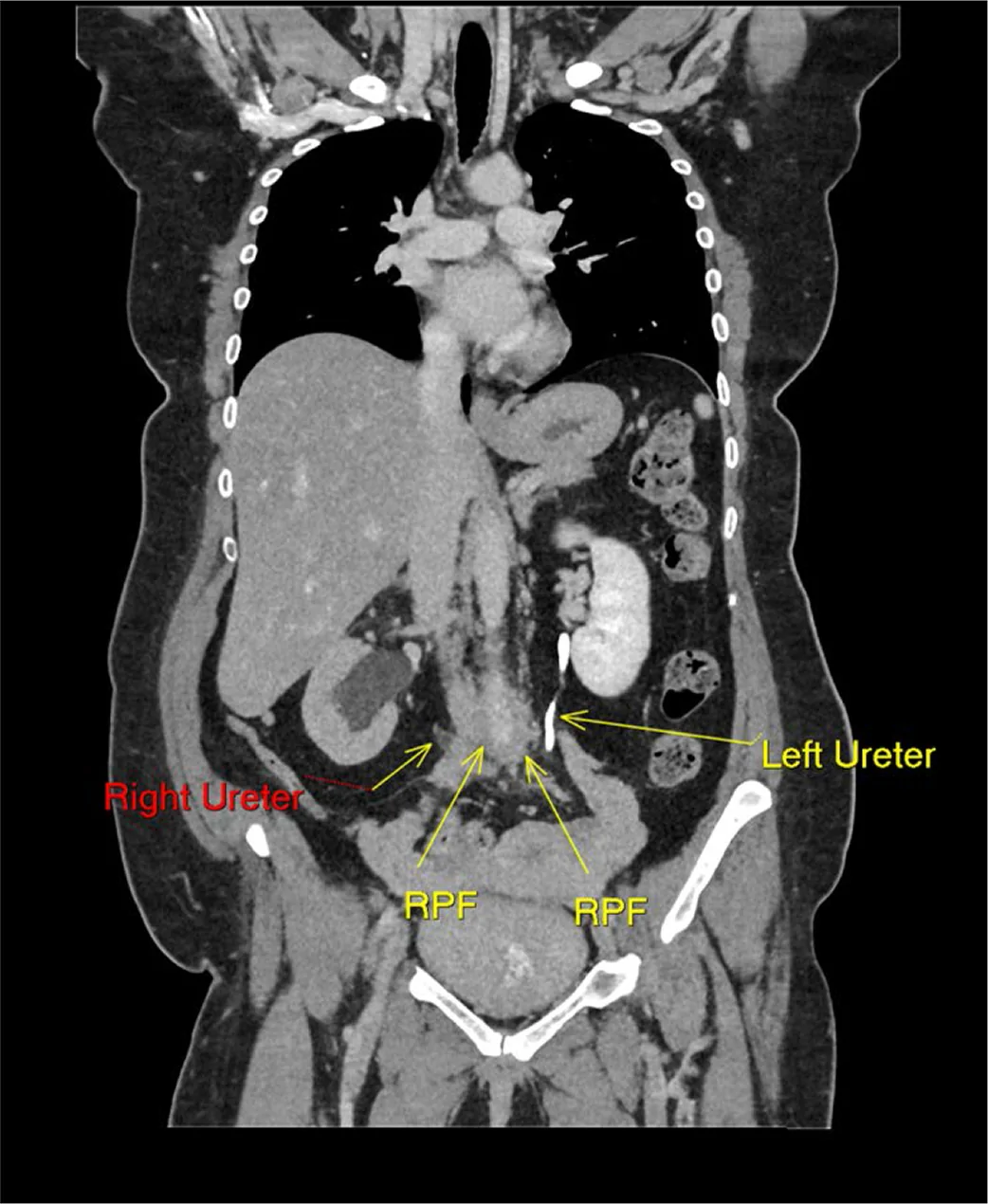
Computer Topography (CT) scan demonstrating retroperitoneal fibrosis.

After seven years of etanercept therapy, the patient presented with recurrent episodes of lower back pain over a 12-month period. Initial magnetic resonance imaging (MRI) findings were suggestive of disc degeneration as the underlying cause. However, she subsequently developed bilateral loin pain, and a computed tomography (CT) scan revealed retroperitoneal soft-tissue thickening encasing the infra-renal abdominal aorta, accompanied by moderate right-sided hydroureteronephrosis (**Figure 1**).

Notably, these findings had not been observed on previous MRI or ultrasound imaging performed over the preceding ten years. The patient experienced a transient rise in serum creatinine from 86 µmol/L to 133 µmol/L. Her IgG4 level was within the normal range, and the protein–creatinine ratio measured at 43 mg/mmol. A right ureteric stent was inserted, followed by nephrostomy. She was commenced on prednisolone 40 mg daily and remained on a dose of 30 mg three months post-procedure. The lesion was not amenable to biopsy, and there was no evidence of lymphadenopathy. The patient’s clinical condition improved, and her ankylosing spondylitis therapy was subsequently switched to bimekizumab, an interleukin-17A/F inhibitor.

IgG4 disease, first described in the year 2000, has been associated with retroperitoneal fibrosis (RPF) and in the described case, similarities could be sought to IgG4 disease however there was a notable absence of lymphadenopathy. Furthermore, the patient’s serum IgG4 levels remained within the normal range, and no other characteristic manifestations, such as orbital, thoracic, or pancreatic involvement, were observed.

A second, emerging entity in the 21st century is etanercept-related RPF. To date, only infliximab and etanercept have been reported with RPF, with no documented cases involving golimumab, adalimumab, or certolizumab use.^6,7^ Mechanistically, infliximab and etanercept are distinct in that they are not fully humanised monoclonal antibodies, which may partly explain this rare paradoxical reaction. Whether interleukin-17 inhibitors possess the potential to induce similar effects remains unclear, as no published literature or reported cases exist of 2025. Through this report, we highlight the potential for IgG4 related disease mimics and the emerging concept of paradoxicality with advances in immunotherapeutics in the 21^st^ century. Further studies are required to understand the underlying mechanisms of PRF and to assess the risk across newer biologic classes.
